# Clinical results of autologous bone augmentation harvested from the mandibular ramus prior to implant placement. An analysis of 104 cases

**DOI:** 10.3205/iprs000100

**Published:** 2016-10-06

**Authors:** Andreas Sakkas, Konstantinidis Ioannis, Karsten Winter, Alexander Schramm, Frank Wilde

**Affiliations:** 1 Department of Oral and Maxillofacial Surgery, University Hospital Ulm, Germany; 2 Department of Oral and Plastic Maxillofacial Surgery, Military Hospital, Ulm, Germany; 3 Department of Prosthodontics, University of Dresden, Germany; 4 Institute of Anatomy, Medical Faculty, University of Leipzig, Germany

**Keywords:** retromolar bone graft, autologous bone, alveolar ridge augmentation, alveolar atrophy, dental implants

## Abstract

**Background:** The aim of this study was the evaluation of the clinical success and complication rates associated with autologous bone grafts harvested from the mandibular ramus for alveolar ridge augmentation and the identification of possible risk factors for graft failure.

**Methods:** In a consecutive retrospective study 86 patients could be included. In these patients a total of 104 bone grafts from the mandibular ramus were harvested for alveolar ridge augmentation. Medical history, age of patient, smoking status, periodontal status and complications were recorded. The need for bone grafting was defined by the impossibility of installing dental implants of adequate length or diameter to fulfill prosthetic requirements, or for aesthetic reasons. The surgical outcome was evaluated concerning complications at the donor or at the recipient site, risk factors associated with the complications and graft survival. All patients were treated using a two-stage technique. In the first operation bone blocks harvested from the retromolar region were placed as lateral or vertical onlay grafts using augmentation templates and were fixed with titanium osteosynthesis screws after exposure of the deficient alveolar ridge. After a healing period of 3–5 months computed tomography scans were performed followed by virtual implant planning and the implants were inserted using guided dental implantation.

**Results:** 97 of the 104 onlay bone grafts were successful. In only 7 patients a graft failure occurred after a postsurgical complication. No long-term nerve damage occurred. Postoperative nerve disturbances were reported by 11 patients and had temporary character only. After the healing period between 4 to 5 months, 155 implants were placed (39 in the maxilla, 116 in the mandible). A final rehabilitation with dental implants was possible in 82 of the 86 patients. Except the 7 graft failures, all recorded complications were minor complications which could be easily treated successfully without any long-term problems. Complications at the donor site were recorded in 3 patients and 17 patients experienced complications at the recipient site. Three of 7 patients with graft failure, were secondarily augmented with a new retromolar graft, harvested from the contra-lateral site and dental implants could be successfully inserted later. No association between complications and smoking habit, age of patient, jaw area, and dental situation (tooth gap or free dental arch) could be detected.

**Conclusions:** Retromolar bone grafts are an effective and safe method for the reconstruction of small- to medium-sized alveolar defects of the maxilla and mandible prior to dental implantation and show a low graft failure rate.

## Zusammenfassung

**Hintergrund:** Ziel dieser Studie war die Evaluation des klinischen Erfolgs sowie der Komplikationsrate nach Alveolarkammaugmentation mit autologen Knochentransplanten aus dem Ramus mandibulae. Des Weiteren galt es mögliche Risikofaktoren für einen Transplantatverlust zu identifizieren.

**Methoden:** In einer konsekutiven retrospektiven Studie wurden 86 Patienten eingeschlossen. Bei diesen Patienten wurden insgesamt 104 Knochentransplantate aus dem Ramus mandibulae zur Alveolarkammaugmentation entnommen. Anamnese, Patientenalter, Rauchverhalten, Parodontalstatus und Komplikationen wurden dokumentiert. Die Indikation zur Knochentransplantation bestand aufgrund eines zu geringen Knochenangebots zur Insertion von dentalen Implantaten in ausreichender Länge oder ausreichendem Durchmesser. Das chirurgische Ergebnis wurde in Bezug auf auftretende Komplikationen in der Entnahme-- oder Empfängerregion, die Risikofaktoren für postoperative Komplikationen und die Transplantat-Überlebensrate evaluiert. Alle Patienten wurden im Rahmen eines zweizeitigen Vorgehens behandelt. Dabei wurden in der ersten Operation retromolare Knochenblöcke entnommen und nach Darstellung des defizitären Alveolarkamms als laterale oder vertikale Onlay-Transplantate unter Verwendung einer Augmentationsschablone mit Mini-Titan-Osteosyntheseschrauben bedarfsgerecht auf dem Alveolarkamm fixiert. Nach einer Einheilzeit von 4–5 Monaten wurden nach computer-assistierter Implantatplanung basierend auf einer Computertomographie die Implantate schablonengeführt inseriert.

**Ergebnisse:** 97 der 104 Knochentransplantate zeigten eine erfolgreiche Einheilung. Bei 7 Patienten kam es aufgrund postoperativer Komplikationen zu einem Transplantatverlust. Eine langfristige Nervenschädigung trat in keinem der Fälle auf. Temporäre postoperative Sensibilitätsstörungen wurden bei 11 Patienten festgestellt. Nach einer Heilungsphase von 4 bis 5 Monaten konnten 155 Implantate inseriert werden (39 im Oberkiefer, 116 im Unterkiefer). Eine Rehabilitation mit dentalen Implantaten war bei 82 der 86 Patienten möglich. Mit Ausnahme der 7 Transplantatverluste konnten alle weiteren Komplikationen ohne Folgen erfolgreich beherrscht werden. Postoperative Komplikationen traten bei 3 Patienten im Bereich der Entnahmestelle und bei 17 Patienten im Bereich der Empfängerstelle auf. Drei der 7 Patienten welche einen Transplantatverlust zu verzeichnen hatten, wurden sekundär mit einem weiteren retromolaren Transplantat aus der kontralateralen Seite augmentiert und konnten in der Folge erfolgreich implantiert werden. Keine der dokumentierten Komplikationen stand statistisch im Zusammenhang mit den Rauchgewohnheiten, dem Patientenalter, der Kieferlokalisation und der dentalen Situation des augmentierten Bereiches (Schaltlücke oder Freiendsituation).

**Schlussfolgerungen:** Die Transplantation von retromolaren Knochenblöcken vor dentaler Implantation erweist sich als eine effektive und sichere Methode zur Rekonstruktion von kleinen bis mittelgroßen Alveolarkammdefekten des Ober- und Unterkiefers bei nur geringem Transplantatverlustrisiko.

## Introduction

The excessive bone loss usually prohibits the placement of dental implants in the ideal prosthetic position. Various techniques and materials have been developed for augmentation of the resorbed alveolar ridge. Based on the volume and density of the existing bone two treatment approaches have been described. In cases with insufficient amount of alveolar bone for primary stability of an implant, the alveolar ridge has to be augmented first before the implant will be placed in a second stage procedure after the bone graft has been incorporated. In cases where the amount of bone allows the achievement of primary stability of the implant, the still exposed threads of the implant can be covered with bone grafts directly after implant placement [1].

Despite of the development of new graft materials, autologous bone still remains as the “gold standard” for bone augmentation procedures because of its osteoinductive, osteoconductive and nonimmunogenic characteristics [2]. Autologous bone can be harvested from extraoral as well as from intraoral donor sites. The great advantages harvesting bone intraoraly is that the surgical procedure can be performed under local anesthesia, lower operative time and costs. The choice of the intraoral donor site is usually based on the amount, geometry and type of bone required for alveolar reconstruction. Additionally, an important factor the surgeon should consider when choosing the donor site, is the incidence of intra- and postoperative complications [3], [4], [5].

In the reconstruction of small alveolar defects, bone grafts from the retromolar region offer several benefits [6], [7], [8], [9]:

1. Proximity of donor and recipient sites that reduces operative and anesthesia time.
2. Conventional uncomplicated surgical access.
3. Minimal discomfort for the patient.

The donor site morbidity and complications after bone harvesting from the retromolar region have been examined by several authors before in prospective and retrospective studies [3], [9]. These clinical studies described minor complications associated with postoperative discomfort of the patient and more serious complications like sensory disturbances of the lower lip. These complications have a negative impact on the patient as well as on the surgeon. The prevention of those complications is very important in order to increase patients’ acceptance of the treatment and compliance. Until now the literature is scarce regarding the factors that affect these complications.

Aims of this study were the evaluation of the clinical success and complication rates associated with autologous bone grafts for alveolar ridge augmentation harvested from the mandibular ramus and the identification of possible risk factors for graft failure.

## Methods

### Patient selection

Patient recruitment and data collection for this study was performed at the department of oral and plastic maxillofacial surgery of the military hospital Ulm, Germany. This research has been conducted in full accordance with ethical principles, including the World Medical Association Declaration of Helsinki. The patient's data was undertaken with the understanding and written consent of each patient and data was also anonymized and de-identified prior to analysis. Reporting was performed based on the recommendations of the Strengthening the Reporting of Observational studies in Epidemiology (STROBE) initiative [10].

For this study we reviewed the records of all patients without exclusion criteria attending our clinic between January 2010 and December 2011 for lateral or bilateral autologous bone augmentation from the retromolar region prior to implant placement. All patients underwent primary clinical and radiographic examination and were diagnosed as having an inadequate quantity of bone for implant placement. 

### Data collection

From the medical files of the patients the following data were collected:

- Medical history of patient
- Age of patient at the time of bone harvesting
- History of periodontal disease
- Smoking status
- Site of augmentation
- Dental situation at the site of augmentation (tooth gap or free dental arch)
- Intra-operative complications
- Post-operative complications
- Management of complications
- Date of implant placement

The indication for augmentation of the alveolar ridge defect was evaluated on the basis of a careful clinical examination with oral inspection and the use of dental casts and a radiological examination using panoramic radiographs to observe the height of the alveolar ridge and to identify structures of risk like the mandibular canal or the maxillary sinus. All the patients were informed in advance that bone grafting was necessary prior to implant placement.

We used a standardized two-stage surgical protocol. In the first intervention, a bone block harvested from the retromolar region was fixed with osteosynthesis titanium screws to the recipient site as onlay graft, to achieve a horizontal enlargement of the alveolar ridge. Placement of the bone graft was always guided by an augmentation template as described by Schramm and Gellrich [11]. In the second procedure, 3–4 months later, the screws were removed and implants were placed using surgical guides based on computer-assisted virtual planning with CoDiagnostiX software (Dental Wings, Canada) [11]. 

The recorded results regarding bone augmentation during the postoperative healing period contained:

- Donor and recipient site morbidity
- Postoperative complications (soft tissue dehiscence, wound infection/abscess formation, graft exposure, hypoesthesia of the mandibular and lingual nerve)
- Bone graft stability
- Bone resorption prior to implant placement

All surgical procedures were carried out under local anesthesia and all sites were treated in a similar fashion. The number of bone blocks, donor sites and number of implants inserted in each augmented site were also recorded. The choice of donor site, left or right was determined preoperatively based on defect morphology and recipient site location. When the augmentation was planned in the posterior mandible one single surgical field for harvesting and transplanting the bone block was opened to decrease patients’ discomfort.

### Surgical protocol

#### Stage 1 surgery

The bone harvesting procedure was performed using a standardized surgical technique. The anesthesia of all patients was carried out with Ultracain^TM^ D-S (Hoechst Marion Roussel Deutschland, Frankfurt, Germany) containing 1:200.000 epinephrine at the donor and recipient sites. A single shot of 2.2 g amoxicillin with clavulanic acid (Augmentan^®^, Glaxo SmithKline Consumer Healthcare GmbH & Co. KG) as well as 250 mg prednisolon (Solu-Decortin^®^, Merck Pharma GmbH) was administered intravenously to patients a few minutes prior to surgery. In cases of penicillin allergy, 600 mg clindamycin (Clinda-saar^®^, MIP Pharma GmbH) was used instead for amoxicillin.

**Figure 1:**
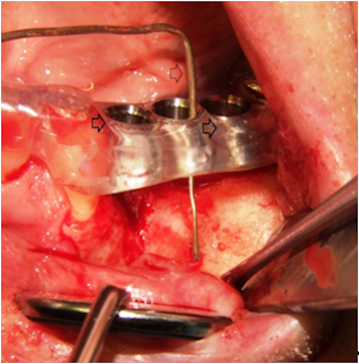
The figure is showing the left maxilla with alveolar ridge atrophy in region 24 to 26 (FDI tooth numbering system). A splint with integrated tubes which are indicating the planed implant position (black arrows) is fixed on the remaining dentition [11]. The probe (white arrow) is indicating the position and amount of bone which is needed for later sufficient implant placement.

The proposed recipient site for the graft was exposed prior to graft harvest in all cases. In this manner, the dimensions and morphology of the bony defect were measured, and minimal time elapsed between graft harvest and placement (Figure 1 (Fig. 1)). 

**Figure 2:**
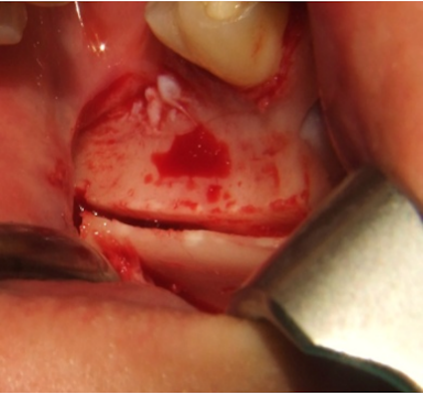
The cranial cut through the cortex is placed on the lateral aspect of the left ramus and the anterior vertical cut was made in the mandibular body in the third molar region in order to harvest the bone graft.

To access the ramus area, the concavity formed by the border between the ascending ramus and the external oblique ridge was identified and used as a starting point for the mucosal incision. The incision was made medial to the external oblique ridge and extended mesially toward the buccal aspect of the second molar. Care was taken to ensure that the incision was not extended too far lingually, preventing damage to structures on the lingual aspect of the mandible. A mucoperiosteal flap was elevated, exposing the lateral aspect of the ramus. The osteotomy was carried out with an osteotomy kit for PIEZOSURGERY^®^ (Mectron, Germany) and was started anterior to the coronoid process at a point with adequate bone thickness. A micro reciprocating saw was used to cut through the cortex along the anterior border of the ramus medial to the external oblique ridge. The anterior vertical cut was made in the mandibular body in the molar region with a vertical saw. The length of this cut was dependent on the size requirements of the graft and on the position of the inferior alveolar canal. The posterior vertical cut was made on the lateral aspect of the ramus, perpendicular to the external oblique osteotomy. The inferior osteotomy connecting the posterior and anterior vertical cuts was made with a straight saw. This was a shallow cut into the ramus to create a line of fracture (Figure 2 (Fig. 2)). 

A thin chisel was gently tapped along the entire length of the external oblique osteotomy and care was taken to parallel the flat site of the chisel with the lateral surface of the bone block, so that a fracture occurred at an exact level avoiding fragmentation of the graft. This level was able to be modified to predetermine the size of the bone graft. The bone block was carefully lifted to ensure that the inferior alveolar nerve was not trapped within the graft. The donor area was filled with a collagen cotton sponge for local hemostasis. 

**Figure 3:**
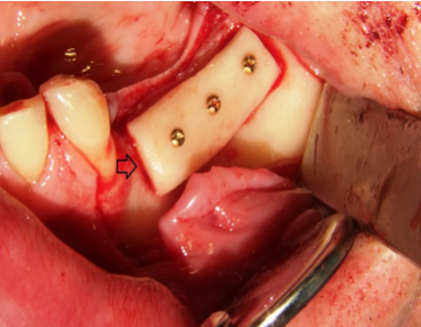
The bone graft is placed in the bone defect in the maxillary left posterior area and fixed with three titanium osteosynthesis screws (arrow).

The block grafts were then fixed with 1.0 mm small-diameter titanium osteosynthesis screws. Additionally, bone chips, which were harvested by using a bone scraper at the donor site as well, were packed around the bone block to fill gaps between the block graft and the recipient bone (Figure 3 (Fig. 3)). Before wound closure with 4/0 resorbable sutures, the entire graft was covered by a collagen membrane (Bio-Gide, Geistlich Biomaterials, Wolhusen, Switzerland). 

Postoperatively, patients were instructed to rinse their mouth with Chlorhexidine 0.2% for 2 to 3 weeks twice daily. After this period the sutures were removed. Removable, strict tooth borne, provisional prostheses were adjusted. Patients were instructed to use their provisional prostheses to eat for the whole period of healing. After three to four months patients were scheduled for implant surgery. 

No antibiotic therapy was continued after surgery and patients were instructed to use non-steroidal anti-inflammatory drugs (Ibuprofen, Docpharm^®^ Arzneimittelvertrieb GmbH & Co. KGaA) only if pain was present. 

#### Stage 2 surgery

After a healing period varying from 4 to 5 months after the grafting procedure, clinical and radiographic evaluations were performed and implants were placed following the implant manufacturers’ surgical protocol. A crestal incision and subperiosteal dissection of the alveolar crest were performed and the fixation screws were removed. Implant site preparation and implant insertion was performed by the use of laboratory manufactured surgical guides.

### Management of postoperative complications

In the case of a postoperative complication due to infection, the complication was managed as follows. Minor effects were treated conservatively with chlorhexidine mouth rinse (0.2%) and antibiotics either oral or intravenous (Augmentan^®^, GlaxoSmithKline Consumer Healthcare GmbH & Co. KG). Patients with wound infection or abscess formation had to be treated surgically in combination with antibiotic cover. By exposed grafts, the surgical field was reopened, the bone block was surgical refreshed with a diamond burr and the flap was tensionless closed in combination with the application of antibiotics for one week.

### Statistical analysis

Statistical analysis included descriptive statistics using SAS^®^ Software Version 9.3. Patient characteristics and complication rates were presented descriptively. As “complication” was considered any adverse event occurred during or after the augmentation procedure (Table 2). To evaluate the association between complication rates and the variables listed below, Chi-square test and Fisher’s exact test were performed. Values of p≤0.05 were considered significant and values of p≤0.005 highly significant. 

Variables:

- smoking status (yes/no)
- age (<40 years, ≥40 years)
- upper or lower jaw
- tooth gap or free-end dental arch

In addition, the results were analyzed in percentage terms and presented in tables and diagrams.

## Results

In 86 patients a total of 104 retromolar bone graft procedures were conducted. Totally, 162 dental areas have been grafted. Patient characteristics are listed in Table 1 (Tab. 1). Mean age of the patients at the time of bone harvesting was 37.9 years. 22 (25.5%) of the patients were smokers and rest of them were nonsmokers. Seven patients (8.1%) were diagnosed with general-advanced periodontitis (according to the American Academy of Periodontology), which was successfully treated before bone grafting. One patient had diabetes mellitus type II. 

22 bone harvesting procedures were performed for augmentation of the maxilla and 82 for the mandible. Regarding the alveolar crest situation 37 cases were recorded as free-end dental arch, 39 as single tooth-gap and 28 as tooth gap more than one tooth.

97 of the 104 retromolar bone grafts were successful. Only seven bone grafts out of the 104 (6.7%) patients have been lost. They were all located in a single tooth-gap dental region and seven different patients were affected. Three of these patients with total graft failure were secondarily augmented with a new retromolar graft, harvested from the contra-lateral site and three dental implants could be successfully inserted later. The remaining four patients wished no further surgical procedure and were treated with a conventional prosthetical restoration. The number of re-augmentations was not taken into consideration in the statistical analysis of the initial patient’s collective and these cases were excluded from the presentation of the final surgical outcome.

No long-term persistent nerve damage of the mental or lingual nerve occurred in all patients. By eleven patients (10.4% of the total cases) a temporary hypoesthesia of the mental area was mentioned and three (2.8% of the total cases) of them reported also sensation disturbance in the tongue. By all of these cases of neural dysfunction, the recipient site of the grafts was in the mandible. None of the patients mentioned an isolated hypoesthesia on the lingual area. At time of implant insertion none of these patients reported of persisting neural disturbances. 

In addition to the seven graft failures just minor complications due to post-surgical infection were recorded at the donor site in three patients and at the recipient site in 17 patients. 

Detailed information of all complications is documented in Table 2 (Tab. 2). Totally 155 implants (39 in the maxilla, 116 in the mandible) could be inserted in the 97 successful augmented sites except the three implants in the three re-augmented sites after primary graft loss. In 95 cases the implantation was performed uneventful. In two cases, the implants (one implant each) were inserted with simultaneous augmentation because of partial bone graft resorption. The average healing period after bone harvesting and grafting was 4.53 months. 

All implants were fully osseointegrated at the time of re-entry for implant uncovery. None of the inserted implants was failed due to lack of osseointegration at the time of prosthetical restoration. After the prosthetic rehabilitation, all aspects of oral function were completely re-established in all patients. 

36.3% of the smokers had complications (8 out of 22). The complication rate for the nonsmokers was 18.7% (12 out of 64). A statistical significance between smoking and complication rates could not be found (p≤0.0916). 

The statistical analysis did also not reveal any association between complication rates and patient's age, history of periodontal disease as well as between jaw area (upper/lower jaw) and occurred complications (p≤0.7211 respectively p≤0.6793). Also no correlation between dental situation (tooth gap or free-end dental arch) and postoperative complications was found (p≤0.4058).

## Discussion

In theory harvesting bone from the retromolar region can cause severe complications, like fracture of the mandible [12] or sensorial disturbances of the lingual or mandibular nerve [13]. However, this cannot be confirmed with this study in which none of these severe complications occured in 104 retromolar bone graft procedures. Therefore, it has to be assumed that the risk for these two complications is very low. Despite the fact that harvesting of autologous bone requires usually a second operation and is associated with increased surgical trauma, the autologous bone grafts are the best solution compared with allografts or xenografts for bone augmentation [2].

However, a disadvantage of grafts from the mandibular raumus remains. Only a confined amount of bone can be harvested from this donor site. It has been described that the volume is half of what can be achieved from the mandibular symphysis [14].

The limits of the retromolar area are usually determined by the reduced clinical access and the limited view. In addition the second and third molar teeth as well as the inferior alveolar canal can limit the amount of harvestable bone. 

The complication rates in the present study may seem relatively high at the first sight. Though, it should be considered that every discomfort or adverse event at the donor or recipient site was recorded as “complication” in this study. 

One important issue we could see in our data was that only three complications due to infection occurred in the donor site area. These three wound infections were minor complications which could be managed easily. This result shows that wound infections in the donor site area after harvesting a bone block in the retromolar region are rather low and can be more or less neglected [14], [15], [16]. 

All our other complications (n=17) on the basis to infection were seen at the recipient sites. This complication rate is comparable to other studies [5], [15], [17], [18], [19]. Seven grafting procedures failed completely. Possible reasons for these graft failures could be microbial contamination due to low compliance of the patients, lack of surgical skills and/or experience of the surgeon or just bad luck. This graft failure rate is comparable to other studies as well [15], [18].

However, the complication that causes a great long term discomfort to the patients is sensorial disturbances of the lingual and/or the mandibular nerve. In the present study these kind of complications occurred in 12.7% of the patients. This is similar to the results of Cordaro et al. (2011) [18]. However, Nkenke et al. (2002), Raghoebar et al. (2007) and Khoury and Hanser (2015) reported lower postoperative sensory disturbances [16], [20], [21]. Nevertheless, the sensorial disturbances in our study were only temporary in all patients and never lasted longer than 3 months. Other studies show comparable results subject to the nerve rehabilitation with duration of no longer than 3–5 months [15], [16], [20]. One study reported re-sensation after 12 months [21].

A damage of the alveolaris inferior or lingual nerve is possible through the bone harvesting procedure from the retromolar region, during the surgical manipulation at the recipient site as well as by block anesthesia of the mandibular nerve. To prevent damaging the mandibular nerve, Khoury and Hanser suggested that local buccal and oral infiltration instead of block anesthesia has to be recommended, to warn the surgeon in cases of reaching the nerve [16].

The risk of nerve damage during bone harvesting was probably additional reduced in our study by the use of piezoelectic ultrasonic surgery which offers a safer way of removing hard tissue without damaging soft tissue in comparison to the conventional surgical burrs and is established as a useful tool of harvesting procedures from the ramus [22]. 

However, the exact reasons which caused the temporary nerve disturbances in this study were not comprehensible retrospectively. Nevertheless, all temporary nerve disturbances in this investigation were after an augmentation procedure in the mandible. In contrast there was no nerve disturbance assed after the 39 augmentations in the maxilla. This indicates that a majority of the nerve disturbances in our study were caused by the augmentation procedure itself and not due to the harvest of the bone block from the retromolar region. 

On the basis of our results we conclude that harvesting bone blocks from the retromolar region is a safe procedure with low risk for nerve damages and a low infection rate.

Comparing bone harvesting from the retromolar region with the chin region, the retromolar area seems to be associated with less patient discomfort [13]. Retrospective studies with observation period between 3 to 5 years report that bone harvesting from the chin region is associated with higher neurosensory disturbances [23], [24]. Recent studies have reported that both ramus and symphysis harvesting procedures are well accepted by patients, but that the ramus procedure is generally preferred [3]. 

The present study shows that smokers did not have a higher risk for post-operative complications. This is in contrast to the results of other clinical trials, which are demonstrating that smokers show a higher failure rate and more postoperative complications than non-smokers [5], [25], [26]. Nevertheless, dentists, oral surgeons and treating physicians should urge their patients to quit smoking. Evaluating these facts in an optimal way, will assist dental professionals when augmentation methods combined with implants are planned in tobacco users. It is extremely important that the practitioner clearly understands and is able to convey the spectrum of possible complications and their frequency to his patients.

No correlation between periodontitis and graft failure rate was determined in this study. This is in contrast to other studies as well [16], [20], [27]. Therefore, the authors suggest that it will be still wise to treat patients with periodontitis in advance and keep them under regular recall before augmentation as well as implantation procedures are performed [27]. 

Despite the low observation period all the inserted implants in grafted sites were successful. Reports from clinical studies show that the survival rates of implants placed in augmented sites are comparable to those placed in non-augmented sites [28], [29], [30].

## Conclusion

According to our experience in this study, we conclude that the method of bone grafting with intraoral bone blocks harvested from the retromolar region is an effective and safe method to treat localized defects of the anterior and posterior maxilla and mandible prior to rehabilitation with dental implants and can lead to long-term success. Graft failure rate and complications using retromolar bone blocks are low and the risk for long-term damage of the mandibular and/or lingual nerve can be neglected. 

A longer follow-up is needed to determine the long-term efficiency of the described grafting technique in comparison to other intraoral donor sites. In addition, more prospective long-term trials are needed to evaluate the implant stability and bone resorption after this method of augmentation.

## Notes

### Competing interests

The authors declare that they have no competing interests and no financial interests, either directly or indirectly, in the products listed in the study.

## Figures and Tables

**Table 1:**
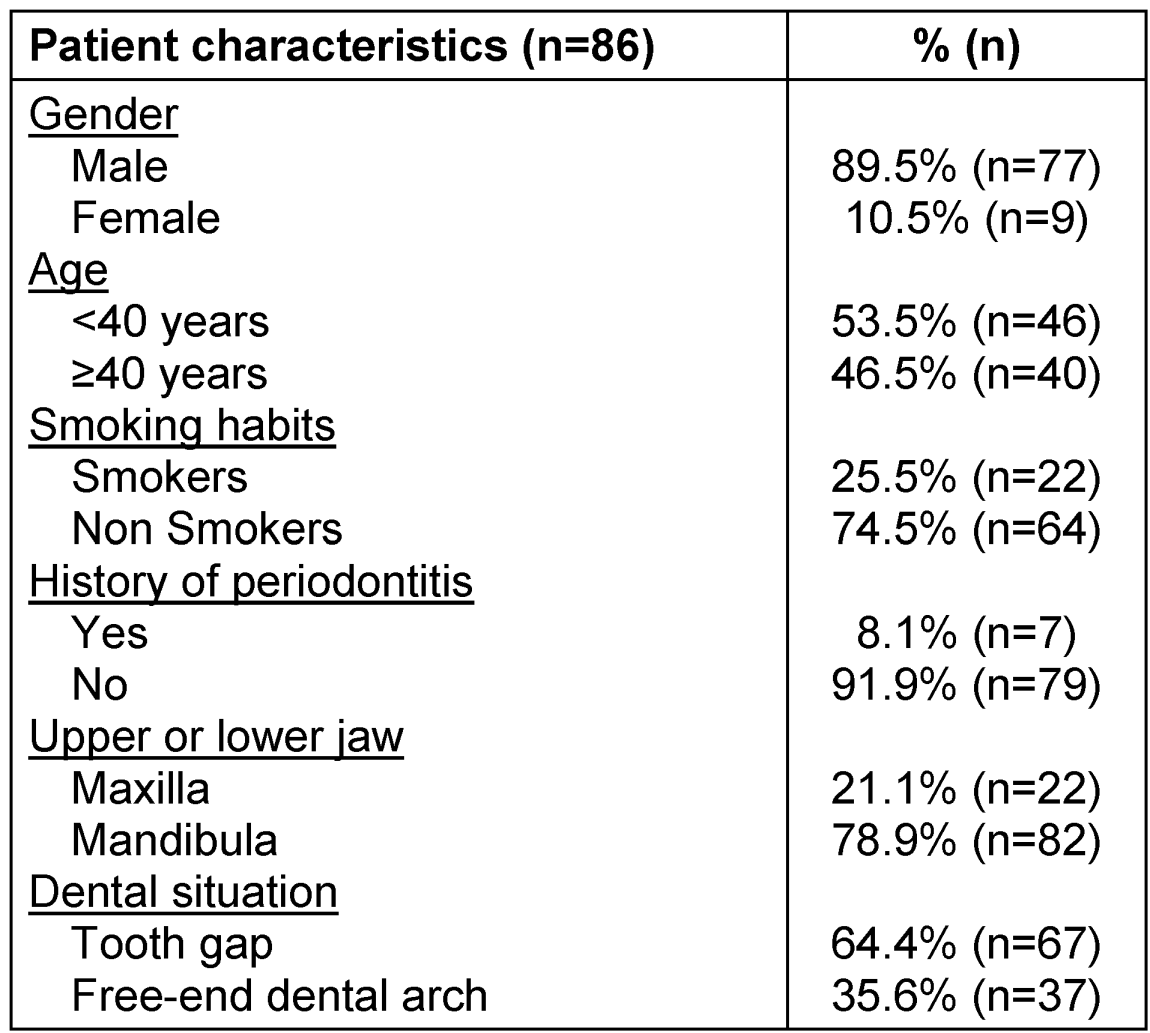
Patient characteristics

**Table 2:**
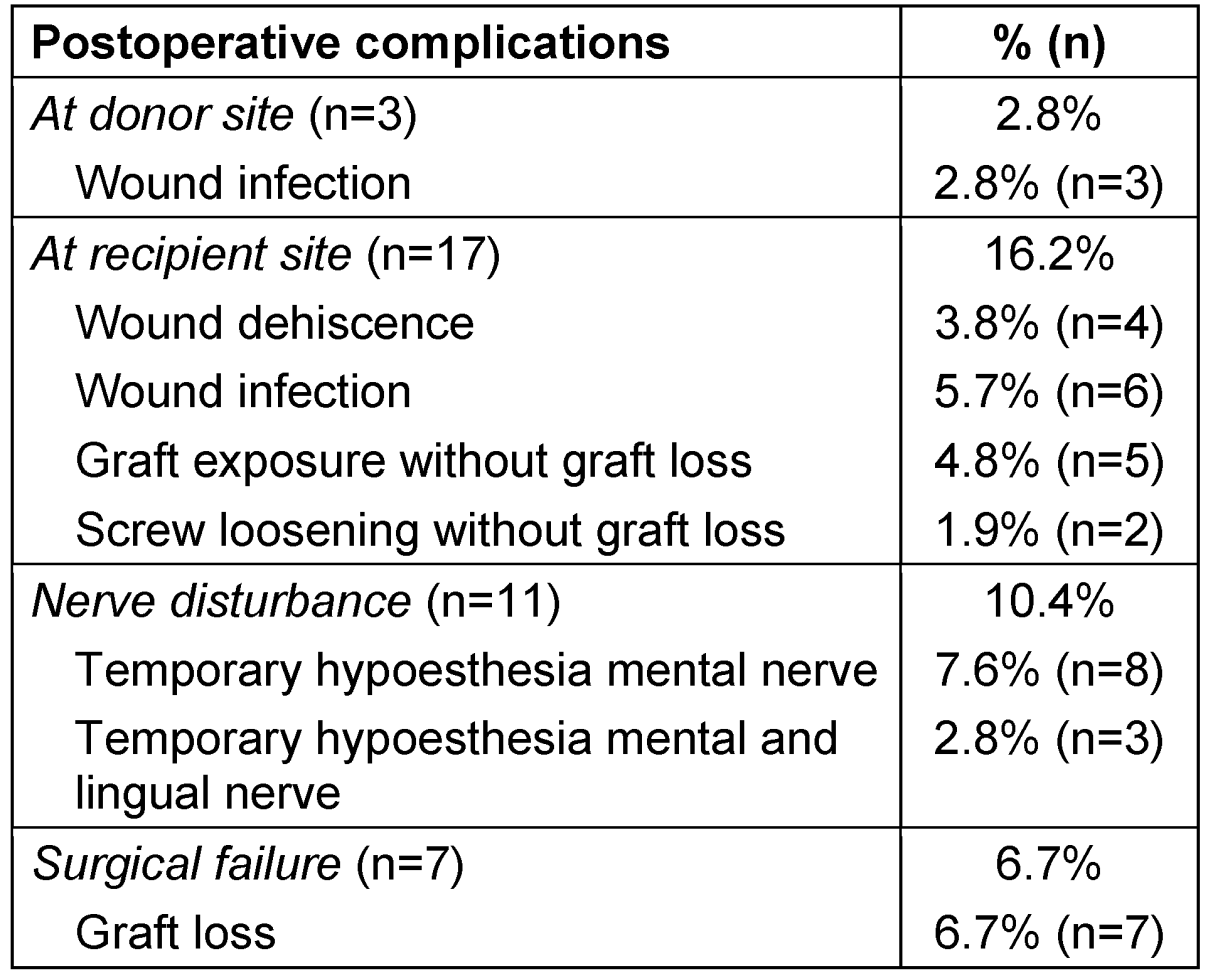
Postoperative complications at the donor and recipient site
